# Kruppel-like factor 13 inhibits cell proliferation of gastric cancer by inducing autophagic degradation of β-catenin

**DOI:** 10.1007/s12672-022-00587-x

**Published:** 2022-11-06

**Authors:** Youxiang Ding, Yuting Xu, Yao Fu, Heng Zhang, Li Zhao, Xiangshan Fan

**Affiliations:** 1 Department of Pathology, Affiliated Drum Tower Hospital, Medical School of Nanjing University, Nanjing, 210008 China; 2 School of Basic Medicine and Clinical Pharmacology, China Pharmaceutical University, Nanjing, 211100 China

**Keywords:** Kruppel-like factor, KLF13, β-catenin, Gastric cancer, Proliferation

## Abstract

Zinc protein KLF13 is a tumor-suppressive member of Kruppel-like factors family, and yet the effect of KLF13 on gastric cancer has not been reported. Here, we aimed to investigate the role of KLF13 in gastric cancer and explored underlying molecular mechanisms. Firstly, it was found that KLF13 expression was significantly decreased in gastric cancer tissues and cancer cells compared with adjacent normal tissues and normal gastric epithelial cells, respectively. KEGG_Pathway and GO_BP analyses suggested that KLF13 was associated with CELL_CYCLE and CELL_PROLIFERATION. Then, our results further demonstrated that KLF13 could obviously inhibit gastric cancer proliferation and induce cell arrest at G2/M phase. Mechanistically, KLF13 decreased expressions of β-catenin and its target genes, CCND1 and MYC, via triggering autophagic degradation of β-catenin. KLF13 up-regulation facilitated co-localization and binding of β-catenin with autophagy protein p62, and exogenous overexpression of β-catenin or blocking autophagy process appeared to reverse KLF13-induced inhibition of gastric cancer proliferation. Furthermore, KLF13 overexpression promoted the expression of ubiquitin-conjugating enzyme E2, Ubc13 which is responsible for catalyzing the synthesis of 'Lys-63'-linked polyubiquitin chains and increased the binding of β-catenin with E3 ubiquitin ligase, TRAF6. In vivo, KLF13 overexpression also suppressed xenograft tumor growth of gastric cancer and down-regulated expressions of Ki67, β-catenin, Cyclin D1, and c-Myc in tumor tissues. Collectively, these data firstly demonstrated the involvement of KLF13 in inhibiting cell proliferation of gastric cancer through promoting autophagy-dependent degradation of β-catenin, which reinforced the evidence for suppressive roles of KLF13 in human tumors.

**Supplementary Information:**

The online version contains supplementary material available at 10.1007/s12672-022-00587-x.

## Introduction

Gastric cancer is one of the most common cancers that threaten people’s lives worldwide. Various risk factors such as *Helicobacter pylori* infection, diets high in nitrates and nitrites, family heredity, lead to initiation and progression of gastric cancer [1]. Although there is a great improvement in treatment efficacy benefiting from surgery, chemotherapy, radiotherapy, immunotherapy, and targeted therapy, overall survival of advanced gastric cancer is still very short [2]. Sustaining proliferative signaling which promotes the chronic proliferation of cancer cells is the principal hallmark of cancer [3]. However, these signal networks are complicated and poorly understood.

β-catenin is a key component of canonical Wnt signaling which not only governs important embryonic and somatic processes, but also plays vital roles in cancers [4]. Aberrant activation of Wnt/β-catenin signaling has been broadly confirmed in cancers and it is involved in diverse cancer processes such as proliferation, apoptosis, metastasis, differentiation, chemoresistance [5]. More specifically, increasing evidence demonstrate that β-catenin accelerates cancer proliferation via mediating multiple signaling transductions and promoting gene transcriptions. It is reported that β-catenin facilitates cell proliferation in diverse cancer types such as hepatocellular carcinoma [6], gastric cancer [7], colorectal cancer [8], breast cancer [9], lung cancer [10], and glioma [11]. Therefore, targeting this signaling has been a potential strategy for cancer therapy [12].

KLF13, also known as BTEB3, FKLF-2, and RFLAT-1, is a ‘repressor’ member of Kruppel-like factors (KLFs) family, which is composed of 17 subtypes, namely KLF1-17 and belongs to zinc-finger transcription factor [13–15]. Based on their transcription activity, KLFs are divided into two sub-family. Consequently, KLF1, 2, and 4–7 function as ‘activator’ while the others act as ‘repressor’ [16]. As a suppressive transcription factor, KLF13 has been found to be involved in some cell processes of several cancer types. KLF13 expression was inhibited in prostate cancer while its overexpression could restrain cell proliferation of prostate cancer through depressing AKT activation [17]. Yao et al. demonstrated that KLF13 was also down-regulated in colorectal cancer and exogenous overexpression of this gene inhibited growth of colorectal cancer via suppressing HMHCS1-mediated cholesterol biosynthesis [16]. Besides, Wu et al. revealed that KLF13 turned to restrain glioma cell invasion and proliferation while AKT activation rescued the effect [18]. Nevertheless, the roles that KLF13 plays in gastric cancer remain unclear.

In this paper, we investigated the effect of KLF13 on gastric cancer proliferation and explored underlying mechanism. As a result, KLF13 inhibited gastric cancer proliferation by triggering the autophagic degradation of β-catenin and reduced expressions of Cyclin D1 and c-Myc. These data firstly revealed the role of KLF13 in gastric cancer proliferation and provided a further understanding of the action mechanism of KLF13 on cancer development.

## Materials and methods

### Cell culture and collection of gastric cancer slides

BGC-823, SGC-7901 and HEK-293 T cell lines were purchased from Shanghai Institute of Cell Biology (Shanghai, China). BGC-823 and SGC-7901 cells were cultured in RPMI-1640 medium (Gibco, USA) while HEK-293 T cells were cultured in Dulbecco’s modified Eagle’s medium (Gibco). All medium was added with 10% fetal bovine serum (Gibco), 100 U/mL benzyl penicillin, and 100 μg/mL streptomycin. All cell lines were incubated at 37 ℃ in a humidified atmosphere containing 5% CO_2_. The paraffin-embedded slides of gastric cancer were provided by Nanjing Drum Tower Hospital. The protocol was approved by The Institutional Ethical Committee of Nanjing Drum Tower Hospital.

### Construct of stable cell line and plasmid transfection

pLenti-CMV-KLF13-GFP/Puro and pLenti-CMV-GFP/Puro were bought from Public Protein/Plasmid Library (Nanjing, China). Lentiviral Packaging Kit (Yeasen, Shanghai, China) was employed to obtain virus supernatants from HEK-293 T cells according to manufacturer’s instruction. Then, gastric cancer cells were infected by the supernatants to construct cell lines with KLF13 overexpression according to the previous method [19]. KLF13 and Empty Vector (EV) plasmids were ordered from Vigene Biosciences (Shandong, China) and Lipofectamine 2000 (Invitrogen, Texas, USA) was used to transfect plasmids into cancer cells.

### CCK-8 assay

5000 population/well of gastric cancer cells were seeded into 96-well plates and cell viabilities after transfection for the indicated times were detected by Cell Counting Kit (CCK-8) (Yeasen) according to the manufacturer’s instruction. OD values were the average absorbances of at least three duplicates.

### Colony formation assay

Colony formation assay was conducted as described previously [20]. Briefly, after being transfected with KLF13 or EV plasmid, 500 cells were seeded into 12-well plates and cultured for 10 days. Then, colonies were fixed, stained, and quantified.

### Cell cycle detection

2 × 10^5^ population of gastric cancer cells were seeded into 12-well plates. After being dealt with the indicated treatment for 24 h, cell suspensions were prepared, and Cell Cycle Analysis Kit (Yeasen) was used to analyze the percentages of cell cycle distribution according to the manufacturer’s instruction.

### Western blot

Western blot analysis was performed according to previous description [19]. Primary antibodies for p62, LC3-I/II, β-catenin, KLF13, β-actin, Cyclin D1, and c-Myc were purchased from WUHAN SANYING (Wuhan, China). Primary antibodies for K63 and K48-linkage Ubiquitin, Ubc13 and TRAF6 were bought from Abcam (Shanghai). HRP Goat Anti-Rabbit IgG (H + L) or HRP Goat Anti-Mouse IgG (H + L) was bought from WUHAN SANYING.

### RT-qPCR assay

TRIzol Reagent (Vazyme, Nanjing, China) was used to extract total RNA. 1 μg RNA was reverse transcribed into cDNA using reverse transcriptase kit (Vazyme). Quantitative real-time PCR assays were performed using ABI7500 (Applied Biosystems) in 8 strips caps (Axygen, Corning, USA) with the SYBR Green PCR Master Mix (Rox, Vazyme). The fold changes were calculated using the 2^−△△Ct^ method. Glyceraldehyde-3-phosphate dehydrogenase (GAPDH) was used as a reference gene. All the primer sequences were presented as follows: *GAPDH* forward 5′-GTCATCCCTGAGCTGAACGG-3′, reverse 5′-AAGTGGTCGTTGAGGGCAAT-3′; *CTNNB1* forward 5′- CACAAGCAGAGTGCTGAAGGTG-3′, reverse 5′- GATTCCTGAGAGTCCAAAGACAG-3′; *CCND1* forward 5′-GATGCCAACCTCCTCAACGA-3′, reverse 5′-GGAAGCGGTCCAGGTAGTTC-3′; *MYC* forward 5′-CGTCCTCGGATTCTCTGCTC-3′, reverse 5′-GCTGCGTAGTTGTGCTGATG-3′.

### Immunofluorescence (IF)

Gastric cancer cells were seeded onto cover glasses and transfected with EV or KLF13 plasmid for 24 h. Then, cells were fixed, permeabilized, blocked, and incubated with anti-p62 or anti-β-catenin antibody respectively. Next day, cells were incubated with Alexa Fluor conjugated secondary antibodies and stained by DAPI. Confocal microscope (Olympus, Tokyo, Japan) was employed to photograph the protein locations in cancer cells.

### Co-Immunoprecipitation (Co-IP)

Cell lysates were incubated with anti‐IgG and Protein A/G PLUS‐Agarose (Santa Cruz, USA). After eliminating beads by centrifugation, anti-β-catenin antibody and beads were added into cell lysates to rotate overnight at 4 °C. Samples were centrifugated, and supernatants were carefully discarded. PBS was used to wash pellet three times, and samples were mixed with 20 μL 2 × loading buffer and boiled. Finally, samples were analyzed by Western blot assay.

### Immunohistochemistry (IHC)

IHC assay was performed according to the previous method [19]. Briefly, paraffin-embedded sections were dewaxed and proceeded with antigen retrieval. After permeabilization and blocking nonspecific sites, primary antibodies were incubated overnight at 4 °C. Next day, DAB solution was used to stain the sections. Finally, sections were stained by HE and covered by neutral gum.

### Bioinformatic analysis

To explore the correlation of KLF13 expression with gastric cancer prognosis, Kaplan–Meier Plotter [21] was employed. Briefly, “KLF13” gene was input and plots of overall survival (OS), first progression (FP), and post-progression survival (PPS) were obtained respectively for gastric cancer. Besides, STRING website was used to explore the potential genes that interact directly or indirectly with KLF13. KEGG_Pathway and GO_BP analyses were performed on Sangerbox website (http://vip.sangerbox.com/login.html).

### Xenograft tumor model

5 × 10^6^ of pLenti-CMV-KLF13 and pLenti-CMV-Vector cells were harvested and injected subcutaneously into armpits of five-weeks old female BALB/c nude mice. Tumor volume (TV) was measured every 2 days and calculated according to the formula: TV = D/2 × d^2^, where D and d are the longest and shortest diameters, respectively. Three weeks later, mice were sacrificed, and tumors were taken out, fixed, and embedded by paraffin.

### Statistics analysis

Each experiment was repeated at least three times. Data values were analyzed using GraphPad Prism 9 software (GraphPad Software, San Diego, CA). Comparisons between two groups were done with Student’s t-test. The data were shown as means ± standard deviation (SD). *P* values < 0.05 were considered statistically significant.

## Results

### KLF13 expression was inhibited in gastric cancer and correlated with gastric cancer prognosis

**Fig. 1:**
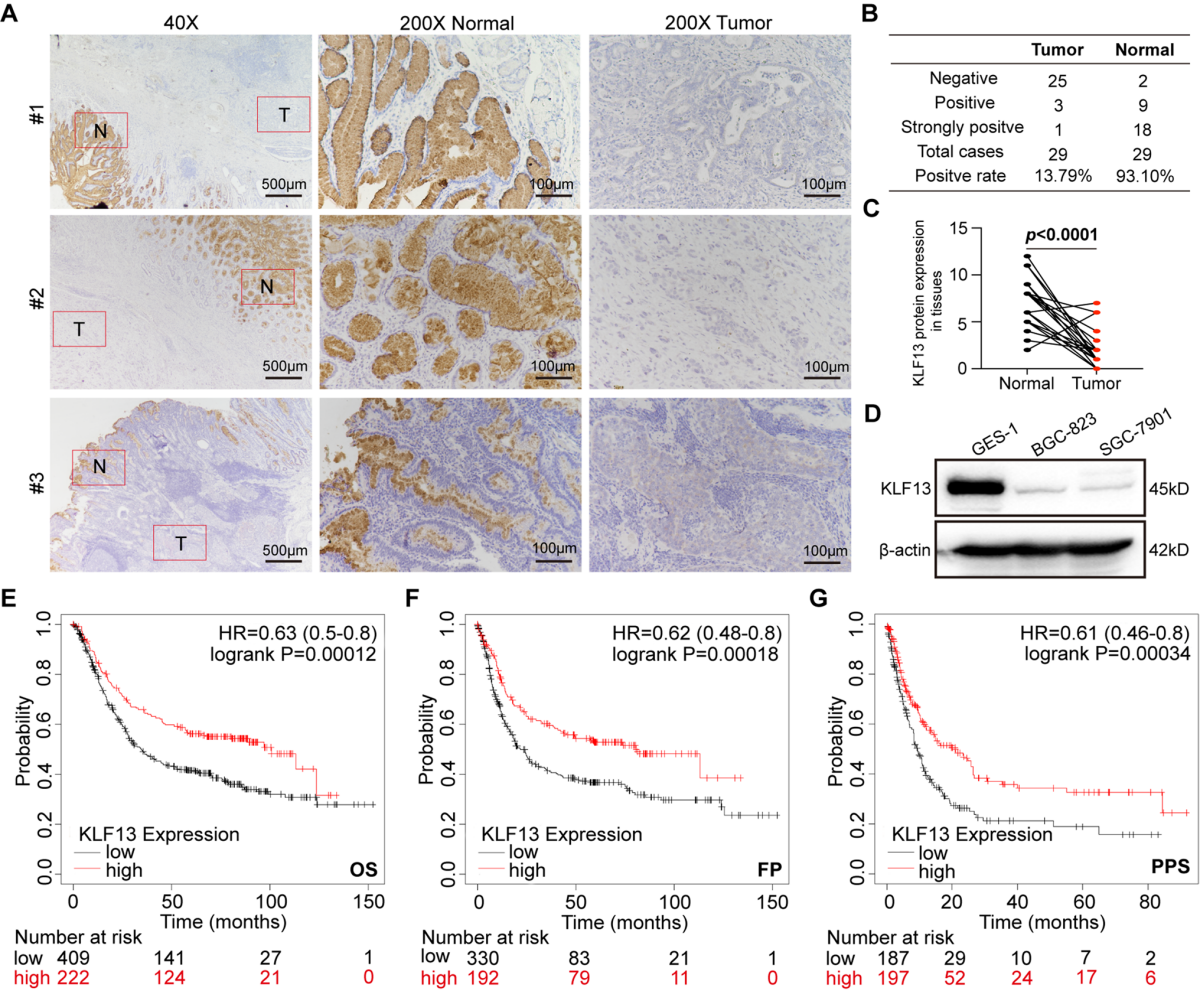
KLF13 expression pattern and its correlation with gastric cancer prognosis. **A** Immunohistochemistry analysis of KLF13 expression in gastric cancer tissues and para-cancer tissues. **B** Statistical analysis of KLF13 expression. **C** Quantification of KLF13 expression in gastric cancer tissues and para-cancer tissues. **D** KLF13 expression in normal gastric epithelial cells and cancer cells. **E**–**G** The correlations of KLF13 expression with the Overall Survival (OS), First Progression (FP), and Post-progression Survival (PPS) of gastric cancer were analyzed using combined datasets, including GSE14210, GSE15459, GSE22377, GSE29272, GSE51105, and GSE62254 via Kaplan–Meier Plotter website

Firstly, we collected 29 pairs of tumor tissues and adjacent normal tissues, and immunohistochemistry assay was conducted to reveal the expression pattern of KLF13 in gastric cancer. As shown in Fig. 1A, it was observed that KLF13 was highly expressed in tissues of gastric pit. However, KLF13 expression in gastric cancer tissues was quite weak or even lost. Statistically, the positive ratio of KLF13 expression in tumor tissues was only 13.79% while this ratio in normal tissues reached to 93.10% (Fig. 1B). KLF13 expression in tumor tissues was significantly lower than that in normal tissues (Fig. 1C). Meanwhile, we also detected KLF13 expression in normal gastric epithelial cells and gastric cancer cells. Western blot analysis indicated that KLF13 expression was strong in normal gastric epithelial cell line GES-1 while it was quite weak in two cancer cell lines BGC-823 and SGC-7901 (Fig. 1D).

Then, Kaplan–Meier Plotter website was employed to analyze correlation of KLF13 expression with prognosis of gastric cancer. As a result, KLF13 expression level positively contributed to a better prognosis of gastric cancer. Briefly, higher KLF13 expression indicated a longer time of overall survival (OS), first progression (FP), and post-progression survival (PPS) in gastric cancer (Fig. 1E−G). Collectively, our results suggested that KLF13 expression was inhibited with the tumorigenesis of gastric cancer, and it might function as a suppressive factor in gastric cancer.

### KLF13 inhibited cell proliferation of gastric cancer in vitro

**Fig. 2:**
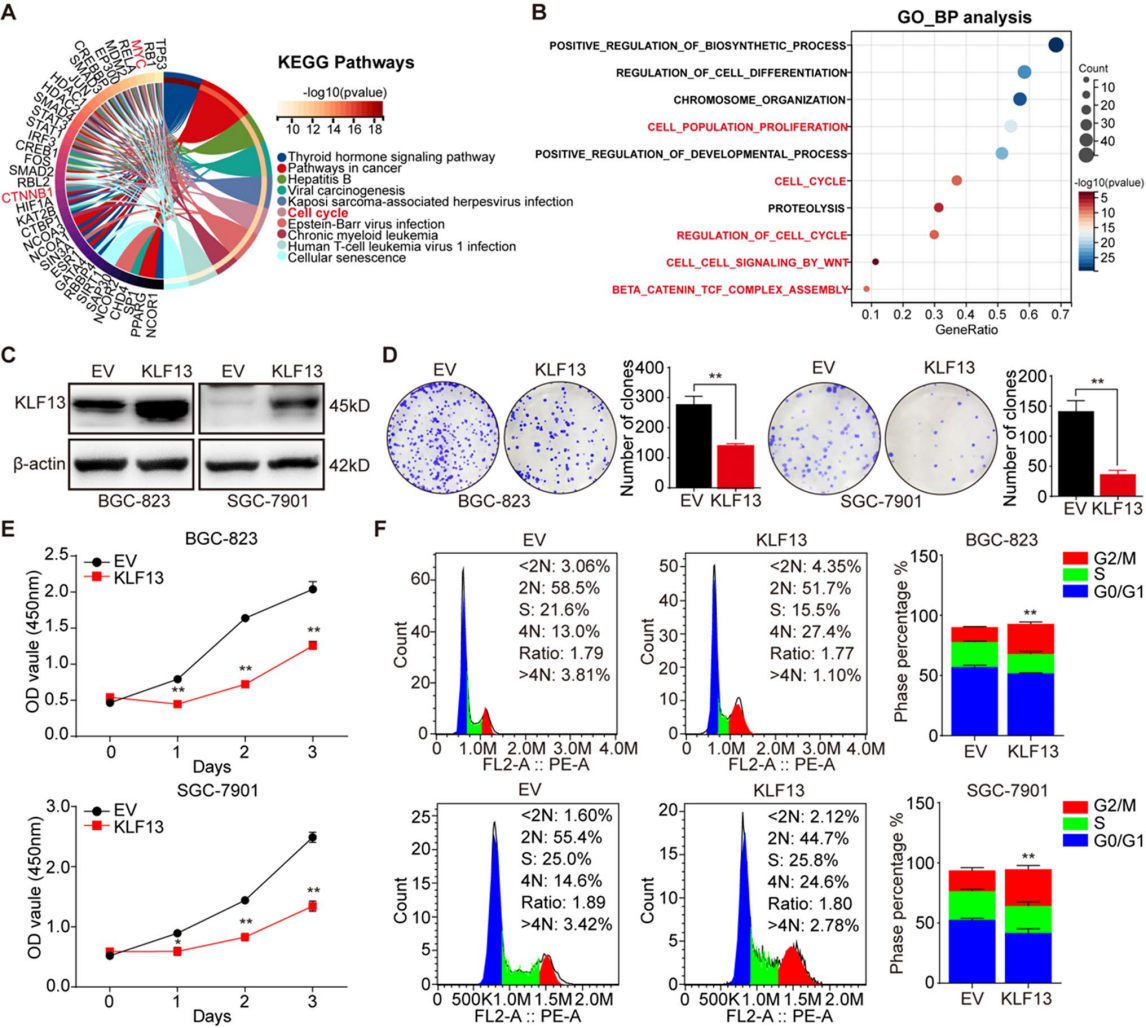
KLF13 induced cell arrest and inhibited proliferation of gastric cancer in vitro. **A**, **B** KEGG pathway and GO_BP analyses of genes that interact directly or indirectly with KLF13. **C** Western blot was conducted to detect the efficiency of KLF13 overexpression in gastric cancer cell lines. **D** Colony formation assay was performed to investigate the effect of KLF13 on cell proliferation. **E** Cell viabilities at the indicated time points were shown using CCK-8. **F** Cell cycle distribution was analyzed through flow cytometry analysis using Propidium Iodide (PI) dye. *P < 0.05, **P < 0.01

To reveal signaling pathways and biological processes in which KLF13 is involved, we sought for some candidate genes that directly or indirectly interact with KLF13 via digging into STRING website and about 71 genes were obtained. Then, we performed the analyses of KEGG_pathway and GO_Biological Process using these genes, and we found that KLF13-related genes were associated with Cell_cycle pathway (Fig. 2A). Meanwhile, GO_BP analysis suggested that these genes were involved in biological processes of CELL_PROLIFERATION, CELL_CYCLE and REGULATION_OF_CELL_CYCLE (Fig. 2B). Collectively, these results reminded us that KLF13 might affect cell proliferation and cell cycle in gastric cancer.

Therefore, to further confirm the effect of KLF13 on gastric cancer, plasmid expressing KLF13 gene was transfected into two gastric cancer cell lines, BGC-823 and SGC-7901, and transfection efficiency was detected by Western blot in Fig. 2C. Then, colony formation assay, CCK-8 assay, and cell cycle analysis using flow cytometry were performed respectively to test capability of cell proliferation of the indicated gastric cancer cell lines which were transfected with KLF13 plasmid. Results in Fig. 2D suggested that KLF13 overexpression could obviously inhibit cell proliferation of BGC-823 and SGC-7901. Meanwhile, results of colony formation assay also indicated that compared with empty vector (EV) group, KLF13 overexpression was found to suppress cell viability in time-dependent manner (0, 24, 48, 72 h) (Fig. 2E).

Cell cycle regulation is essential for cell proliferation and deregulation of cell cycle underlies the uncontrolled cell proliferation that is characterized by malignant tumor [22]. Therefore, we also investigated the changes of cell cycle distribution by using flow cytometry in cells transfected with KLF13 plasmid or not. As shown in Fig. 2F, KLF13 overexpression turned out to induce cell arrest at G2/M phase in both gastric cancer cell lines. Taken together, these data revealed that KLF13 played a suppressive role in gastric cancer and its overexpression could inhibit cell proliferation and induce cell arrest at G2/M phase. However, there is no evidence reporting the underlying molecular mechanism by which KLF13 restrains the proliferation of gastric cancer.

### KLF13 suppressed β-catenin expression and transcription of its downstream genes, CCND1 and MYC

**Fig. 3:**
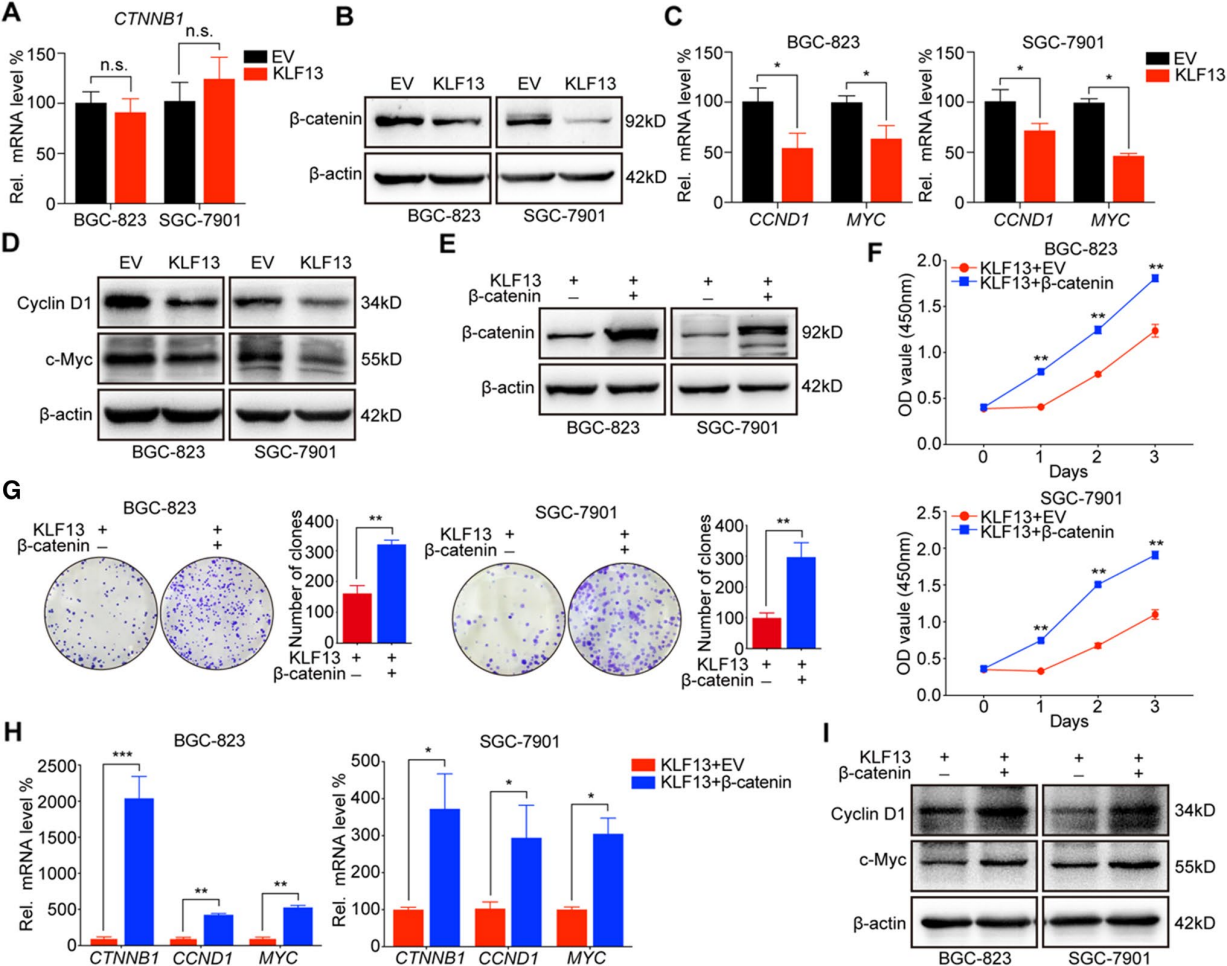
KLF13 suppressed cell proliferation via inhibiting β-catenin expression and its downstream genes, CCND1 and MYC. **A**, **B** Effect of KLF13 on the expression of β-catenin was analyzed by RT-qPCR and Western blot assays, respectively. **C** mRNA levels of CCND1 and MYC were detected by RT-qPCR assay. **D** Protein expressions of cyclin D1 and c-Myc were evaluated via Western blot. **E** KLF13 plasmid alone or with plasmid expressing CTNNB1 gene was transfected into gastric cell lines and β-catenin expression was observed by Western blot. **F**, **G** CCK-8 and Colony formation assays were conducted to evaluate the effects of β-catenin overexpression on KLF13-mediated inhibition of cell proliferation. **H**, **I** The reversal effects of β-catenin overexpression on KLF13-induced downregulations of CCND1 and MYC expression were analyzed by RT-qPCR and Western blot, respectively. n.s.: not significant, *P < 0.05, **P < 0.01, ***P < 0.001

β-catenin is a key component of Wnt signaling and it plays an important role in regulating cell proliferation and survival through promoting gene transcription [23]. KEGG pathway and GO_BP analyses in Fig. 2A, B indicated that KLF13 correlated with CTNNB1 and MYC, and concurrently, KLF13 seemed to regulate biological processes of CELL_CELL_SIGNALING_BY_WNT and BETA_CATENIN_TCF_COMPLEX_ASSEMBLY. Consequently, we investigated whether KLF13 could affect β-catenin to inhibit cell proliferation in gastric cancer. RT-qPCR and Western blot were conducted to clarify the effect of KLF13 overexpression on β-catenin expression at mRNA and protein levels, respectively. It was found that KLF13 had no significant effect on mRNA level of CTNNB1 which encodes β-catenin (Fig. 3A). However, β-catenin expression at protein level was obviously reduced in gastric cancer cells transfected with KLF13 plasmid (Fig. 3B), which revealed that KLF13 might regulate β-catenin expression via translation or post-translation process instead of transcription level.

CCND1 and MYC are two typical target genes of β-catenin and these genes play an important role in cancer proliferation [7]. Thus, we further analyzed the expression changes of two genes by RT-qPCR and Western blot, respectively. Our results indicated that KLF13 could remarkably decrease both expressions of CCND1 and MYC at mRNA and protein levels (Fig. 3C, D). Based on these results, we speculated that β-catenin-dependent CCND1 and MYC transcriptions appeared to be involved in KLF13-induced suppression of gastric cancer proliferation. To further demonstrate this speculation, we exogenically overexpressed β-catenin in gastric cancer cells transfected with KLF13 plasmid (Fig. 3E) and analyzed expressions of CCDN1 and MYC, along with viability of cell proliferation. Results of CCK-8 and colony formation assay demonstrated that cell proliferation of BGC-823 and SGC-7901 was boosted after plasmid expressing CTNNB1 was transfected into cells in the presence of KLF13 overexpression (Fig. 3F, G). Moreover, analyses of Western blot and RT-qPCR suggested that β-catenin overexpression was able to rescue KLF13-mediated down-regulation of CCND1 and MYC expressions in both gastric cancer cell lines (Fig. 3H, I).

Moreover, we also analyzed on-line ChIP-sequencing data using several available websites such as PROMO, Cistrome DB, and JASPAR to detect whether Cyclin D1 and c-Myc could be direct transcriptional targets of KLF13. However, all data indicated that there was no binding site in the promoter regions of both Cyclin D1 and c-Myc for KLF13 (data were not shown), which demonstrated that these two genes were not direct transcriptional targets of KLF13.These data revealed that KLF13 suppressed cell proliferation through inhibiting β-catenin expression and its downstream genes, CCND1 and MYC, in gastric cancer.

### KLF13 inhibited β-catenin expression via triggering the autophagic degradation of β-catenin

**Fig. 4:**
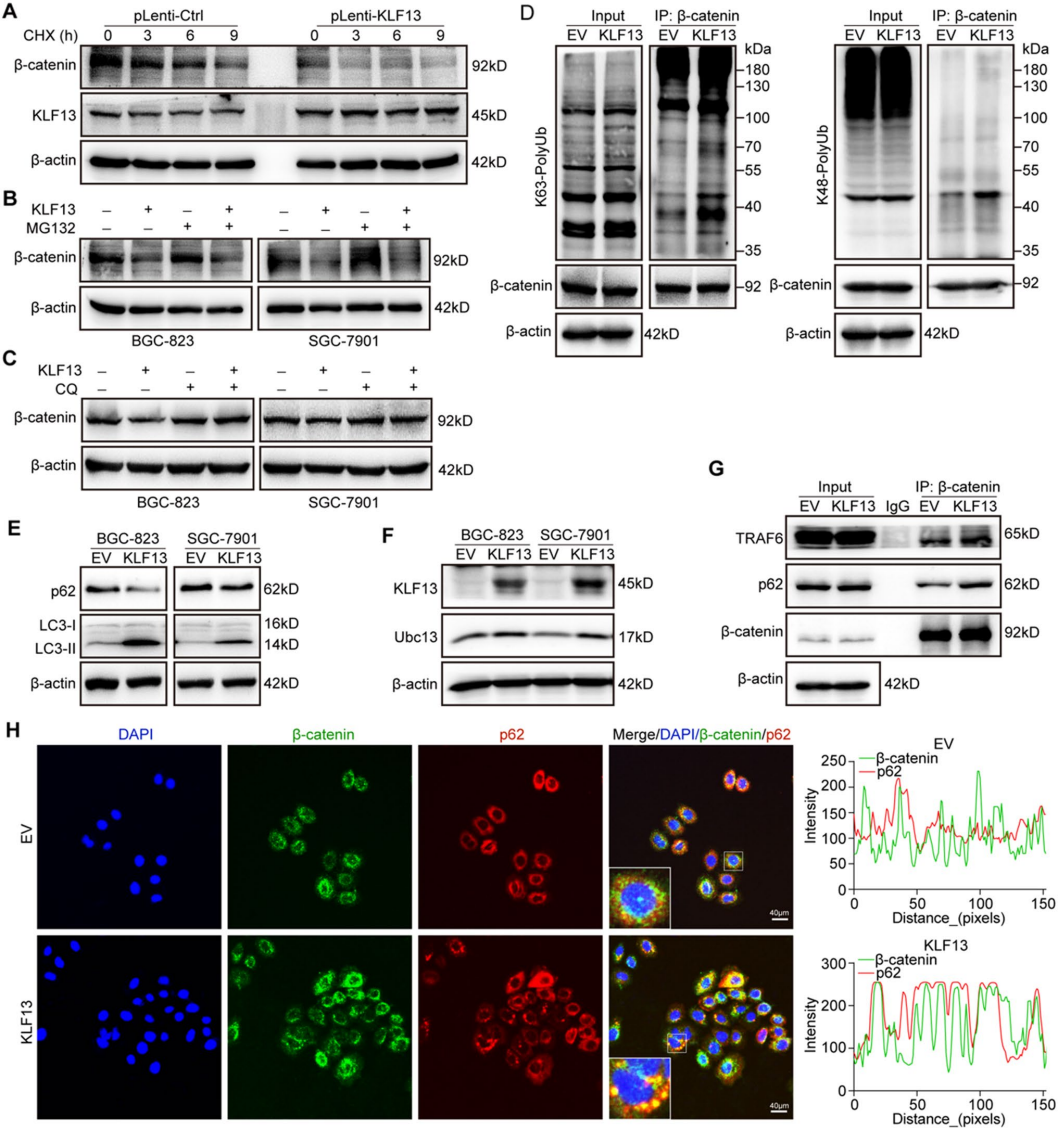
KLF13 decreased β-catenin expression in autophagic degradation manner. **A** pLenti-Ctrl and pLenti-KLF13 of BGC-823 cell lines were treated with 10 μg/mL CHX for the indicated times and β-catenin stability was analyzed by western blot. **B**, **C** 10 μM MG132 and 10 μM CQ were employed respectively to reveal the involvements of proteasome and autophagy in KLF13-induced degradation of β-catenin. **D** Co-IP assay was conducted to analyze the K63-linkage and K48-linkage poly-ubiquitylation of β-catenin in BGC-823 cells transfected with KLF13 plasmid or not. **E** Two key biomarkers of autophagy, namely p62 and LC3-I/II were detected. **F** Effect of KLF13 overexpression on ubiquitin-conjugating enzyme E2, Ubc13. **F** Binding of β-catenin with E3 ubiquitin ligase, TRAF6 or autophagy protein, p62 was analyzed. **H** Immunofluorescence assay was performed to investigate the effect of KLF13 on the colocalization of β-catenin and p62 in BGC-823 cells. Z-axis profile analysis was presented at right panel. Scale bar = 40 μm

Here, to elucidate mechanism underlying KLF13-mediated inhibition of β-catenin expression at protein level, we explored the involvements of translation process and protein degradation in β-catenin expression by using an inhibitor of protein synthesis, Cycloheximide (CHX) and two inhibitors, MG132 and Chloroquine (CQ) for proteasome-dependent and autophagy-dependent degradation, respectively. After treatment with 10 μg/mL CHX for indicated times, β-catenin expression was down-regulated in time-dependent manner in pLenti-Ctrl group of BGC-823 cells (Fig. 4A). Furthermore, this effect was more notable in pLenti-KLF13 group, which reminded us that KLF13 could inhibit β-catenin expression via promoting its protein degradation. To figure out the manner of β-catenin degradation induced by KLF13, MG132 and CQ were used respectively in gastric cancer cells transfected with KLF13 plasmid or not. Western blot analyses suggested that MG132 failed to reverse KLF13-induced decrease of β-catenin expression in both two cell lines (Fig. 4B). On the contrary, autophagy inhibitor CQ was found to reverse down-regulation of β-catenin expression caused by KLF13 overexpression in gastric cancer cells (Fig. 4C). Therefore, we speculated that KLF13 inhibited β-catenin expression through triggering its autophagic degradation.

K63-specific poly-ubiquitination serves as a recognition signal for multiple autophagy receptors and it is a key modification for autophagic degradation of protein substrate while K48-linkage poly-ubiquitination is responsible for proteasomal degradation [24]. Hence, to further confirm our speculation, we performed a Co-IP assay using K63-sepecific and K48-linkage ubiquitin antibodies to analyze ubiquitin modification type of β-catenin in BGC-823 cells treated with KLF13 plasmid or not. Results in Fig. 4D showed that KLF13 promoted K63-specific modification of β-catenin, which was helpful for the autophagic degradation of β-catenin. However, there was no obvious change for its K48-linkage ubiquitin modification. We further detected autophagy flux via Western blot analyses of LC3-I/II and p62 in gastric cancer cells. As shown in Fig. 4E, KLF13 overexpression could reduce p62 expression and promote the specific expression of LC3-II subtype in both two cell lines, which suggested that KLF13 triggered the autophagy flux in gastric cancer cells. Meanwhile, we also investigated the effect of KLF13 on colocalization and binding of β-catenin with autophagy protein p62, and it was observed that KLF13 promoted colocalization and binding of two proteins in BGC-823 cells (Fig. 4G, H). More colocalization puncta were observed in BGC-823 cells with KLF13 overexpression. These data demonstrated that KLF13 inhibited β-catenin expression via triggering the autophagic degradation of β-catenin.

Ubc13, also known as Ubiquitin-Conjugating Enzyme E2N (UBE2N), is responsible for catalyzing the synthesis of 'Lys-63'-linked polyubiquitin chains [25]. TNF Receptor Associated Factor 6 (TRAF6), a E3 ubiquitin ligase, can cooperate with UBE2N and UBE2V1 to mediate the synthesis of 'Lys-63'-linked-polyubiquitin chains conjugated to proteins such as IRAK1, AKT1 and AKT2 [26–28]. Therefore, we studied the effect of KLF13 up-regulation on both Ubc13 and TRAF6 to verify how KLF13 triggered the autophagic degradation of β-catenin. Our results demonstrated that KLF13 overexpression promoted the expression of Ubc13 (Fig. 4F) and increased the interaction of β-catenin with E3 ubiquitin ligase TRAF6 (Fig. 4G). These data provided more evidence for autophagic degradation of β-catenin triggered by KLF13.

### Autolysosome inhibition could rescue KLF13-induced suppression of cell proliferation in gastric cancer in vitro

**Fig. 5:**
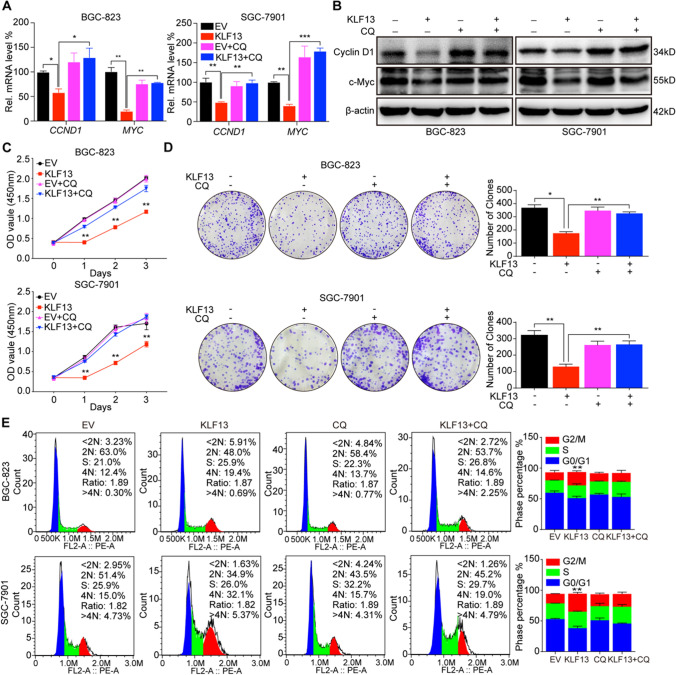
Autolysosome inhibition could rescue KLF13-induced suppression of cell proliferation in gastric cancer in vitro. **A**, **B** Influences of autolysosome inhibition by CQ in KLF13-mediated expressions of CCND1 and MYC were analyzed by RT-qPCR and Western blot. **C**, **D** CCK-8 and Colony formation assays were conducted to observe the effects of CQ on KLF13-induced impairment of cell proliferation. **E** Flow cytometry analysis of cell phase distribution. *P < 0.05, **P < 0.01, ***P < 0.001 compared with empty vector (EV) group if not indicated

To further clarify the involvement of autophagic degradation of β-catenin in KLF13-suppressed cell proliferation of gastric cancer, we detected the effects of autophagy inhibition on expressions of CCND1 and MYC as well as cell proliferation by adding CQ into the cells transfected with KLF13 plasmid or not. Analyses of RT-qPCR and Western blot found that CQ reversed KLF13-induced down-regulation of CCND1 and MYC at both mRNA and protein levels (Fig. 5A, B). Besides, results of CCK-8 and colony formation assay showed that CQ could rescue the viability of cell proliferation inhibited by KLF13 overexpression in BGC-823 and SGC-7901 cell lines (Fig. 5C, D). Moreover, KLF13 was demonstrated to induce cell arrest at G2/M phase in gastric cancer cells, and thus we also analyzed whether CQ could reverse this effect. Unsurprisingly, autophagy inhibition by CQ could significantly reduce G2/M arrest in both cell lines of gastric cancer (Fig. 5E). All these data further demonstrated that KLF13 suppressed cell proliferation through promoting β-catenin degradation in autophagy-dependent manner in gastric cancer in vitro.

### KLF13 restrained gastric cancer proliferation in vivo through decreasing β-catenin expression

**Fig. 6:**
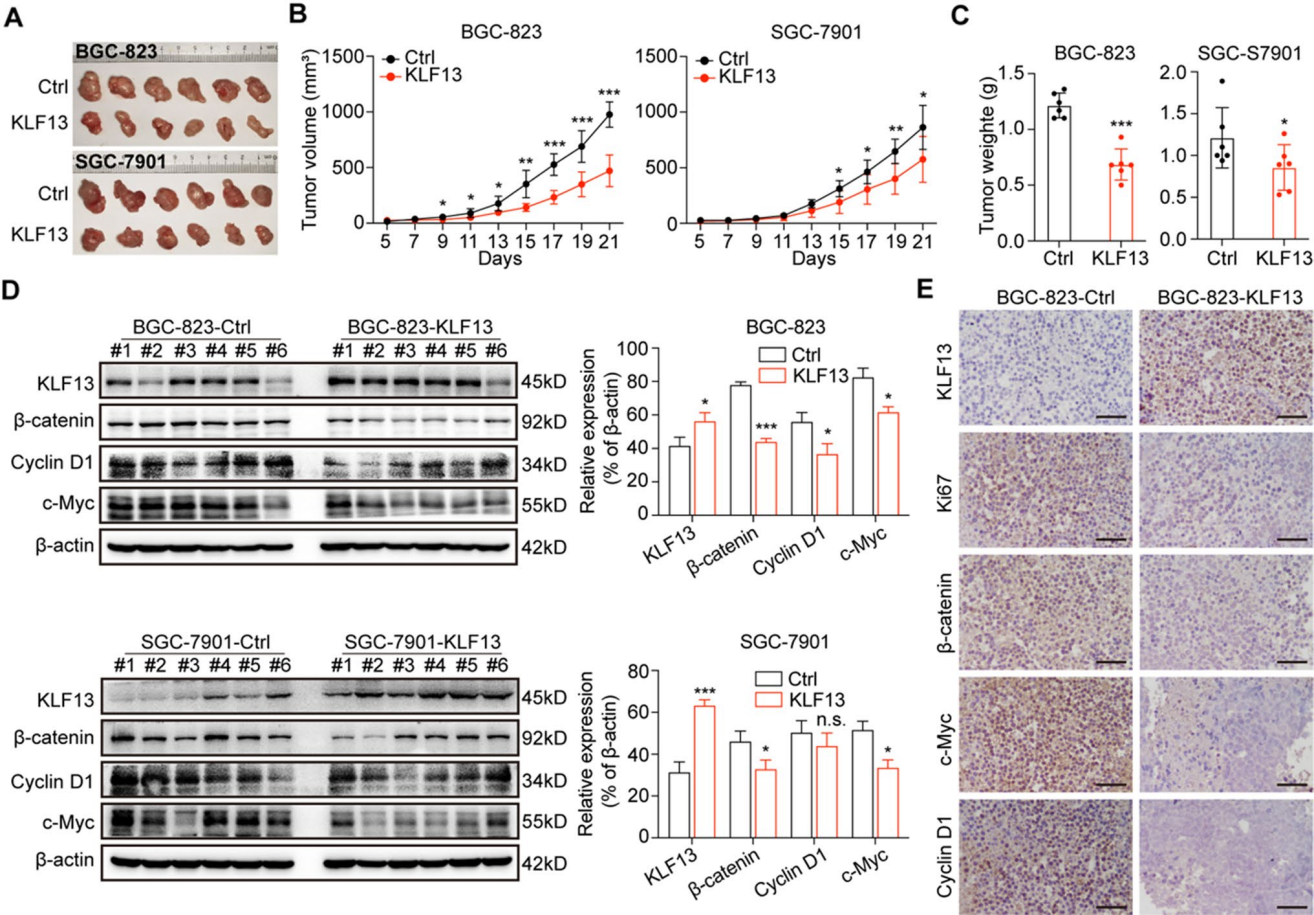
KLF13 inhibited β-catenin expression and suppressed gastric cancer proliferation in vivo. **A** Tumor photos of the indicated groups of BGC-823 and SGC-7901 cells. **B** Tumor growth plots of BGC-823 and SGC-7901 cell lines with KLF13 overexpression or not. **C** Tumor weight statistics of the indicated groups of two gastric cancer cell lines. **D** Western blot was employed to investigate the effect of KLF13 overexpression on the expressions of β-catenin and its downstream, Cyclin D1 and c-Myc in xenograft tumors. **E** Immunohistochemistry stainings of KLF13, Ki67, β-catenin, c-Myc, and Cyclin D1 of the indicated groups were presented. Scale bar = 50 μm. n.s.: not significant, *P < 0.05, **P < 0.01, ***P < 0.001

To elucidate the effect of KLF13 on gastric cancer growth in vivo, we constructed a xenograft tumor model using female BALB/c nude mice with stable-transfection cell lines of BGC-823 and SGC-7901. Our results showed that KLF13 overexpression obviously inhibited tumor growth of gastric cancer compared to the corresponding control group in both cell lines (Fig. 6A, B). Meanwhile, the tumor weights of KLF13 groups were significantly smaller than those of control groups in both cell lines (Fig. 6C). These data indicated that KLF13 also restrained gastric cancer proliferation in vivo. Then, we continued to investigate whether KLF13 could.

decrease β-catenin expression and the expressions of its target genes, CCND1 and MYC, in xenograft model. Western blot analyses of tissue homogenates showed that KLF13 overexpression could significantly reduce protein expressions of β-catenin, Cyclin D1 and c-Myc in most mice (Fig. 6D). Besides, results of IHC assay also indicated that KLF13 obviously inhibited Ki67 expression, along with down-regulating the protein expressions of β-catenin and its downstream Cyclin D1 and c-Myc in tumor tissues (Fig. 6E). Taken together, KLF13 also restrained gastric cancer proliferation through decreasing β-catenin expression in vivo.

## Discussion

As a ‘repressor’ member of KLFs family, there is no evidence reporting the role of KLF13 in gastric cancer. In this paper, immunohistochemistry analysis uncovered that KLF13 was specifically and notably expressed in tissues of gastric pit while it was quite weak or even lost in tumor tissues. Moreover, we firstly found that KLF13 overexpression could suppress gastric cancer proliferation both in vitro and in vivo. Mechanistically, KLF13 decrease expressions of β-catenin and its target genes CCND1 and MYC via triggering autophagic degradation of β-catenin. Although some research data, including ours and others, suggest that KLF13 suppresses cell proliferation in some cancer types, the effects of KLF13 on cancer initiation and progression are still poorly understood. Studies concerning the involvements of KLF13 in different cancer processes such as metastasis, differentiation, inflammation, angiogenesis, chemoresistance, immune response, are few. Therefore, more efforts are supposed to be made to reveal the functions of KLF13 in tumorigenesis along with clarifying the underlying molecular mechanisms.

Actually, there are many evidences indicating that KLF13 is associated with some non-neoplastic diseases such as microdeletion syndrome [29], cardiac abnormality [30], HPV productive life cycle [31], adipocyte differentiation [32], pulmonary fibrosis [33], non-alcoholic fatty liver disease [34]. Also, KLF13 is found to contribute to some processes of heart development [35–37]. Some reports point out that KLF13 is also involved in immune-related regulations via affecting the development and function of immune cells [38–40]. These evidence indicate that KLF13 is versatile and it has multiple functions in regulating diverse physiology processes of non-neoplasia and tumor.

Sustaining proliferation is a vital characteristic of cancer and it is necessary for tumor initiation and progression. This biological process is activated by the increasing activity and expression of cell cycle-related proteins as well as some signal transduction pathways [41]. Consequently, it has become a key standard care by targeting signaling pathways which contribute to uncontrolled tumor proliferation for the patients [42]. Although an increasing number of small molecular inhibitors as well as natural compounds have been developed to inhibit cancer proliferation, the treatment efficacy is far to be satisfying. Signaling networks that promote the chronic proliferation of tumor cells are remarkably complicated and our understanding of molecular mechanisms responsible for cancer proliferation is still poor. Besides, these signals are not independent, and they have been cross-talking with other signaling networks to facilitate tumor initiation and development. Therefore, a treatment regime by only inhibiting one molecular target which accelerates cancer proliferation is often found ineffective.

## Conclusion

In this paper, we found that KLF13 overexpression inhibited gastric cancer proliferation both in vitro and in vivo. Mechanistically, KLF13 down-regulated β-catenin expression through triggering the autophagic degradation of β-catenin. As a result, expressions of two target genes of β-catenin, namely CCND1 and MYC, which are important for cancer proliferation, were suppressed. Collectively, our results firstly demonstrate the role of KLF13 in gastric cancer proliferation and provide a further understanding of the molecular mechanism underlying this process.

## Supplementary Information

**Additional file 1.** Original images of western blots.

## Data Availability

All data used or analyzed during the current study are available from the corresponding author on reasonable request.
