# Supplementary figures and images for: Kruppel-like factor 13 inhibits cell proliferation of gastric cancer by inducing autophagic degradation of β-catenin
Source: Discov Oncol. 2022 Nov 6;13:121. doi: 10.1007/s12672-022-00587-x (PMC9637683; doi:10.1007/s12672-022-00587-x)

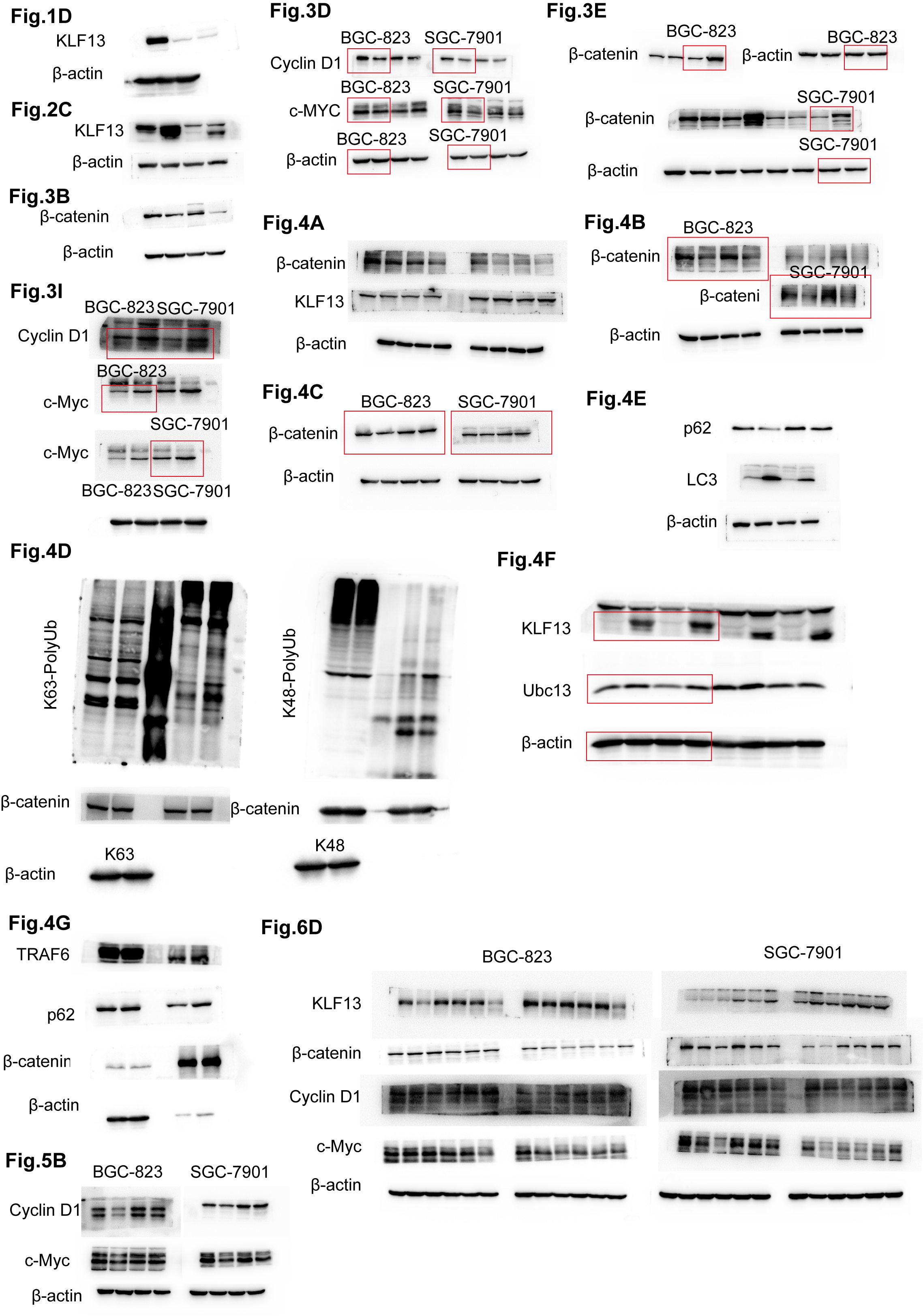

Supplement: Supplementary file 1 — Additional file 1. Original images of western blots. [file 12672_2022_587_MOESM1_ESM.tif]
